# AHCC^®^ Supplementation to Support Immune Function to Clear Persistent Human Papillomavirus Infections

**DOI:** 10.3389/fonc.2022.881902

**Published:** 2022-06-22

**Authors:** Judith A. Smith, Anjali A. Gaikwad, Lata Mathew, Barbara Rech, Jonathan P. Faro, Joseph A. Lucci, Yu Bai, Randall J. Olsen, Teresa T. Byrd

**Affiliations:** ^1^Department of Obstetrics, Gynecology and Reproductive Sciences, UT Health McGovern Medical School, Houston, TX, United States; ^2^Department of Pharmacy, UT Heath-Memorial Hermann Cancer Center, Houston, TX, United States; ^3^UT Physicians Women’s Center, Houston, TX, United States; ^4^Specialists in Obstetrics & Gynecology, Houston, TX, United States; ^5^Department of Pathology, UT Health McGovern Medical School, Houston, TX, United States; ^6^Department of Molecular Pathology, Houston Methodist Research Institute, Houston, TX, United States

**Keywords:** AHCC, cancer prevention, cervical cancer, HPV, immunomodulation, interferon-beta, nutritional supplements

## Abstract

**Objective:**

To determine the efficacy, safety, and durability of the use of AHCC supplementation for 6 months to support the host immune system to clear high-risk human papillomavirus (HPV) infections. The AHCC supplement is a proprietary, standardized extract of cultured lentinula edodes mycelia (AHCC^®^, Amino Up, Ltd., Sapporo, Japan) that has been shown to have unique immune modulatory benefits.

**Study Design:**

This was a randomized, double-blind, placebo-controlled study (CTN: NCT02405533) in 50 women over 30 years of age with confirmed persistent high-risk HPV infections for greater than 2 years. Patients were randomized to placebo once daily for 12 months (N = 25) or AHCC 3-g supplementation by mouth once daily on empty stomach for 6 months followed by 6 months of placebo (N = 25). Every 3 months, patients were evaluated with HPV DNA and HPV RNA testing as well as a blood sample collected to evaluate a panel of immune markers including interferon-alpha, interferon-beta (IFN-β), interferon-gamma (IFN-γ), IgG1, T lymphocytes, and natural killer (NK) cell levels. At the completion of the 12-month study period, patients on the placebo arm were given the option to continue on the study to receive AHCC supplementation unblinded for 6 months with the same follow-up appointments and testing as the intervention arm.

**Results:**

Fifty women with high-risk HPV were enrolled, and 41 completed the study. Fourteen (63.6%) of the 22 patients in the AHCC supplementation arm were HPV RNA/HPV DNA negative after 6 months, with 64.3% (9/14) achieving a durable response defined as being HPV RNA/HPV DNA negative 6 months off supplementation. On the placebo arm, two (10.5%) of 19 patients were HPV negative at 12 months. In the twelve placebo arm patients who elected to continue on the unblinded study, 50% (n = 6) were HPV RNA/HPV DNA negative after 6 months of AHCC supplementation. At the time of completion of the study, there were a total of 34 patients (22 blinded and 12 unblinded) who had received AHCC supplementation with an overall response rate of 58.8% that cleared HPV persistent infections. At the time of enrollment, the mean IFN-β level was 60.5 ± 37.6 pg/ml in women with confirmed persistent HPV infections. Suppression of IFN-β to less than 20 pg/ml correlated with an increase in T lymphocytes and IFN-γ and durable clearance of HPV infections in women who received AHCC supplementation.

**Conclusion:**

Results from this phase II study demonstrated that AHCC 3 g once daily was effective to support the host immune system to eliminate persistent HPV infections and was well tolerated with no significant adverse side effects reported. The duration of AHCC supplementation required beyond the first negative result needs more evaluation to optimize success for durable outcomes. The suppression of the IFN-β level to less than 20 pg/ml correlated with clearance of HPV infections and merits further evaluation as a clinical tool for monitoring patients with HPV infections.

**Clinical Trial Registration:**

clinicaltrials.gov/ct2/, identifier NCT02405533

## Introduction

The human papillomavirus (HPV) is classified as a non-enveloped, double-stranded DNA virus that generally infects the epithelial layer of cells including cutaneous and mucosal surfaces and is associated with benign warts, carcinoma *in situ*, and malignant lesions (1, 2). There are over 100 HPV strains identified in humans, 40 low-risk HPV (LR-HPV) strains associated with genital warts/lesions, and fifteen high-risk HPV (HR-HPV) strains associated with cancer. When HR-HPV infections persist over time, patients have an increased risk of developing cancer (3). However, it should be noted that having a persistent high-risk HPV infection does not cause cancer by itself; rather, it is a contributing co-factor in the risk for development of cancer when it occurs in combination with other insults such as poor nutrition, smoking, physiological stress, or immune dysfunction/suppression. In the United States, there are an estimated 85,890 cases of cancer caused by persistent high-risk HPV infections, and an estimated 79 million Americans are infected with HPV today (4, 5).

Although HPV vaccination is effective in the prevention of HPV infections, it has little benefit for the treatment of patients already infected with HPV (6). There are very few effective treatment options for eradicating high-risk HPV infections. The objective of current treatment modalities relies upon early detection with routine PAP smear screening and then treatment by the physician employing cryotherapy, surgical excision, loop electrosurgical excision procedure (LEEP), or cold knife conization. These procedures have a higher response rate of 80% to 100%. This local treatment removes the lesion, but patients will frequently have recurrent lesions. Other treatment modalities used for LR-HPV treatment include topical application of podophyllotoxin with up to 40% rate of recurrent lesions or imiquimod with an approximate 15% recurrence (7, 8). To date, there is no effective systemic treatment for persistent high-risk HPV infections.

AHCC is a proprietary, standardized extract of cultured lentinula edodes mycelia (AHCC^®^, Amino Up, Ltd., Sapporo, Japan) that was developed in Japan in 1992; the compound is primarily composed of α-glucan components. Several animal and human studies have reported a variety of therapeutic effects, including antioxidant and anticancer activities and modulation of the immune system to prevent the infectious processes of both viral and bacterial infections (9–14). In clinical studies, AHCC has demonstrated the benefit to decrease the risk of infection and ameliorate symptoms of existing infections (11).

Two pilot studies recently were conducted on women who had documented persistent HPV positive for greater than 2 years, were otherwise healthy, and met the remaining eligibility criteria (15). Ten women were enrolled in the first study to evaluate the effectiveness of AHCC supplementation of 3 g by mouth once daily to support the host immune system to eliminate persistent high-risk HPV infections. The regimen was repeated monthly until the patient tests HPV negative or until 6 months of active treatment has elapsed. Patients who test positive after 6 months of treatment were considered a treatment failure. HPV testing and immune marker panel screening were completed once a month to monitor for clearance of high-risk HPV persistent infections. There was an encouraging response in 4 of 8 (50%) patients with confirmed HR-HPV DNA eradication after at least 3 months and up to 6 months of daily 3-g AHCC supplementation (15). In the second pilot study with 1-g AHCC supplementation once daily, a similar response was observed in 4 of 9 (44%) patients with confirmed clearance of high-risk HPV persistent infections after 7 months of supplementation (15). These results from two pilot studies confirm the previous preclinical findings that AHCC supplementation has the potential to be effective to support the host immune system to clear persistent high-risk HPV infections and supported the rationale to continue forward with a formal phase II evaluation to confirm these preliminary findings (16).

## Materials and Methods

### Study Design

**Figure 1:**
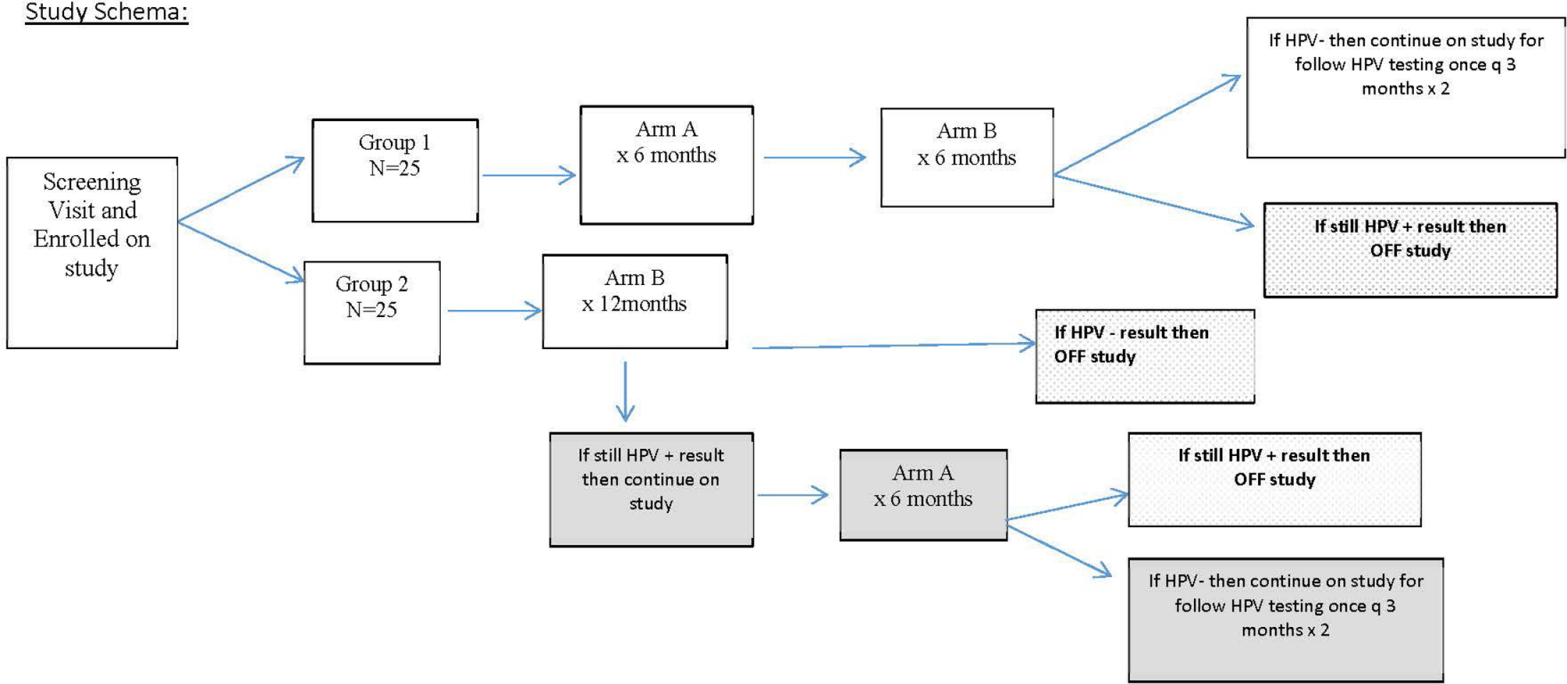
Study design for the enrollment of patients. This schema explains how patients were randomized and extent of follow-up based on human papillomavirus (HPV) response at 12 months.

This was a phase II randomized, double-blind, placebo-controlled study with post-study unblinded AHCC (**Figure 1**) that was reviewed and approved by the University of Texas Health Sciences Center Institutional Review Board HSC-MS14-0866/*NCT02405533*. The study was conducted in women over the age of 30 with documented persistent high-risk HPV infections for over 2 years or more, had laboratory values all within normal limits, had normal histology up to cervical intra-epithelia neoplasia 2 (CIN2) as documented by the primary gynecologist’s records, and deemed otherwise healthy. Patient demographic information was collected including the number of lifetime sexual partners, contraception methods, and periodic pregnancy testing throughout the study. AHCC and matching placebo were generously provided by Amino Up, Ltd. (Sapporo, Japan).

After eligibility was confirmed based on criteria in **Table 1**, patients provided signed informed consent and were randomized fairly *via* computer platform between the two study groups so that after 30 patients were enrolled, there were 15 patients on each arm, and at the completion of the study enrollment, there were 25 patients on each arm. Group 1 served as the intervention arm and received AHCC supplementation of 3 g by mouth on an empty stomach once daily for 6 months followed by 6 months of the placebo by mouth on an empty stomach. The purpose of the 6 months of placebo in group 1 was to help facilitate maintaining blinding through the 12-month study time point. Group 2 served as the control arm and received placebo by mouth on an empty stomach for 12 months.

**Table 1:** Inclusion and exclusion criteria for phase II study.

**Inclusion criteria:** • Women over 30 years of age who have an HPV positive test and normal/negative cytology, atypical cells, ASCUS, or CIN1 or CIN2 cervical dysplasia within 3 months of study entry. This minimized potential confounders such as immune modulation that may possibly clear the infection, which is common in women under the age of 26. • Women must have had 2 other HPV-positive tests with normal/negative cytology, atypical cells, ASCUS, or CIN1 or CIN2 cervical dysplasia ○ 1 greater than 6 months and no more than 18 months prior to study entry and ○ 1 greater than 24 months prior to study entry. (This is to help establish persistent HPV infection.) • Women of childbearing potential must have a negative urine pregnancy test within 7 days of therapy start. • Patients must have adequate hematologic, renal, and hepatic functions as noted from labs within the previous 12 months: ANC ≥ 1,500 cells/mm3, platelets 100,000 ≥cells/mm3; creatinine clearance ≥ 60 ml/min (estimated by Cockcroft Gault equation), total bilirubin, SGPT, SGOT, and alkaline phosphatase ≤ 1.5 times normal. • Patients of childbearing age must agree to use effective methods of contraception (oral contraceptives, condoms, etc.) while on study. • Patients must provide an approved informed consent indicating that they are aware of the investigational nature of this study. • Patients must agree to return to clinic for follow-up visits (including repeat HPV testing) and complete medication administration calendar.	**Exclusion criteria:** • History of myocardial infarction within past 6 months, unstable angina, CHF, or uncontrolled hypertension (>140/90). • Women with a current or prior diagnosis of cancer. • Women with a current diagnosis of CIN3 cervical dysplasia. • Women who are pregnant or breastfeeding. • Women with a history of hepatitis (autoimmune, A, B, or C) or antigen positive. • Patients with history of significant psychiatric disorders (schizophrenia, bipolar, and psychosis) or uncontrolled seizures. • Patients with significant medical comorbidities at the discretion of the primary gynecologist. Including immunosuppressive conditions (i.e., HIV+ and rheumatoid arthritis) or taking immune modulation mediations (i.e., immunosuppressants). • Women who have taken commercial supply of AHCC within the past 6 months on their own. Those who have been participating in the AHCC 1 g day pilot study are eligible to enroll in this study. • Women currently taking other immune-modulating nutritional supplements.

ASCUS, atypical squamous cells of undetermined significance; ANC, absolute neutrophil count; SGPT, serum glutamic pyruvic transaminase; SGOT, serum glutamic-oxaloacetic transaminase; HPV, human papillomavirus; CHF, congestive heart failure.

Patients were monitored once every 3 months during the study with a clinic visit to review the pill diary to assess compliance, evaluate any toxicity experienced over the previous 90 days, and collect HPV samples *via* cervical swab/brush and peripheral blood sample. The study was unblinded at 12 months to determine HPV status and immune marker results and reveal study arm assignments. All patients who received AHCC and were HPV negative at 12 months were monitored for an additional 6 months (for a total of 12 months of monitoring after completion of AHCC supplementation). At 12 months, if on placebo the control arm patients were still HPV positive, then patients were offered to continue on AHCC unblinded to gain more information on its toxicity and efficacy. The purpose of “crossover” after unblinding time point was to also help enhance recruitment and enrollment since AHCC is a readily available nutritional supplement.

The primary outcome of this trial was to evaluate clearance of persistent high-risk HPV infection determined by HPV DNA-negative test results achieved while receiving AHCC supplementation and maintained for 3, 6, and 12 months post-completion of the AHCC supplementation compared to receiving placebo. At 12 months, after receiving AHCC supplementation for 6 months, if patients were still HPV DNA positive, it was considered a treatment failure or no response (NR), and they went off study. If negative after completion of 6 months of AHCC supplementation and 6 months of placebo, patients continued on study for another 6 months (two visits) to confirm they remained HPV negative and to evaluate the durability of the response. A complete response (CR) was defined as those patients who were HPV RNA and HPV DNA negative at the time of completion of AHCC supplementation and remained HPV RNA and HPV DNA negative throughout the 12 months of follow-up off AHCC supplementation. A partial response (PR) was defined as those patients who were HPV RNA and HPV DNA negative at the time of completion of AHCC supplementation but then tested either HPV RNA or HPV DNA positive at one or more visits in the 12 months of follow-up off AHCC supplementation. Group 2 served as the control for all time points for the duration of the study. Safety and efficacy data were collected in the patients who elected to continue on unblinded AHCC supplementation for 6 months.

### Sample Collection and Processing

At each visit, a gynecologic (cervical or vaginal wall) specimen was collected by three methods—cytology brush, cytology broom, and cervical spatula—to ensure an adequate sample and transferred to a ThinPrep^®^ liquid gynecology specimen vial (Hologic, Inc., Marlborough, MA, USA). Twelve milliliters of whole blood was collected in EDTA (BD Biosciences, San Jose, CA, USA) tubes from all patients at every visit. All blood samples were centrifuged at 1,500 rpm for 10 min, and plasma samples were separated and stored at −80°C within 6 h of collection until further use. Peripheral blood mononuclear cells (PBMCs) were isolated from remaining blood by density gradient centrifugation over Ficoll Lymphocyte Separation Medium (Lonza, Basel, Switzerland) using the manufacturer’s protocol. The PBMCs were cryopreserved in a freezing medium containing 90% fetal bovine serum (FBS) and 10% dimethyl sulfoxide (DMSO) in liquid nitrogen (−196°C) until further use. FBS was purchased from GIBCO Invitrogen Co. (Carlsbad, CA, USA). The DMSO was purchased from Sigma-Aldrich Co. (St. Louis, MO, USA).

#### Human Papillomavirus Sample Analysis

HPV samples were evaluated initially with HPV E6/E7 RNA testing (APTIMA, Hologic, Marlborough, MA, USA) at the UTHealth McGovern Medical School Molecular Pathology laboratory. HPV RNA-negative results were confirmed with COBAS HPV DNA testing (Roche Molecular Systems, Inc., Branchburg, NJ, USA) at Methodist Molecular Pathology laboratory to detect the presence of HPV DNA and rule out latent HPV infections.

#### Immune Marker and Natural Killer Cell Assays

Ready-to-use sandwich ELISA kits for the detection of human Interferon-alpha and beta were purchased from R&D Systems (Minneapolis, MN, USA). Human interferon-gamma (IFN-γ) and IgG1 ELISA kits were purchased from Affymetrix eBioscience (San Diego, CA, USA). Sandwich ELISAs for the detection of total IgG1, interferon-alpha, interferon-beta, and interferon-gamma were performed according to the manufacturer’s protocol. For each series of immune marker determinations, a standard curve was constructed with known concentrations of these markers provided with the kits. Plasma concentrations of these immune markers at each time point were calculated from standard graphs and were compared with baseline concentrations.

Five mouse anti-human antibodies were used for the identification and quantification of human natural killer cells (NK cells): CD56 APC clone, B159; CD3 PerCP-Cy5.5 clone, UCHT1; CD2 PE clone, RPA 2.10; CD7 FITC clone, M-T701; and CD45 APC-H7 clone, 2D1. All antibodies and fluorescence-activated cell sorting (FACS) lysing solutions were purchased from BD Biosciences (San Jose, CA, USA). For flow cytometry analysis of human NK cells, the PBMCs cryopreserved at every time point were gently thawed and centrifuged with 5 ml of a complete medium at 1,500 rpm for 5 min at 4°C. The cell pellets were re-suspended in 100 µl of FACS buffer (phosphate-buffered saline (PBS) with 5% FBS). For staining of the cells, one set of cells from each sample was incubated with 5 µl of CD45 APC, and the second set of cells was incubated with 50 µl of a cocktail of five antibodies for 15 min in the dark at room temperature as per the manufacturer’s protocol. Following the incubation, 1 ml of FACS lysing buffer was added to all tubes and was further incubated for 5 min in the dark at room temperature. To stop the lysis of cells, 1 ml of PBS was added to each tube immediately after incubation. All tubes were then centrifuged at 1,500 rpm for 5 min, and cells were re-suspended in 500 µl of FACS buffer and stored at 4°C.

The stained cells were acquired using LSR II (BD Biosciences), and Diva software was used for analysis. With the use of forward and side scatter, the lymphocyte population was gated on low side scatter and CD45 bright population while acquiring the 50,000 gated events. The NK cell population was then identified as (CD3 negative, CD 2, CD56C, and CD7 positive) cells. The percent of T cells and NK cells from the lymphocyte gate was obtained for the final analysis.

### Statistical Analysis

In this population, women with persistent HPV infections, the expected eradication or clearance of HPV infection on its own is zero to 10%. The target success rate based on pilot study data was 50% at 6 months after the end of AHCC supplementation and 12 months after the end of supplementation. Based on these proportions, 10% clearance in the absence of supplementation and 50% clearance with supplementation, a power analysis was conducted using data simulation in Mplus 7.2 through a multi-step process (17). The proportional difference was expected to be observed between AHCC supplementation intervention group 1 and placebo control group 2 at the end of the 6 months posttreatment. At a 0.05 confidence level, a sample of a maximum of 50 patients (N = 25 per group) has 94.5% power to achieve detection of the effect of the study intervention. Baseline characteristics of patients by study group were compared using the chi-square test for categorical variables and Student’s test for continuous variables. The Mann–Whitney test was used to evaluate the differences in response rates based on the durability of HPV response (NR, PR, and CR) between the study arms that responded versus non-responders. Student’s t-test analysis was used to evaluate the differences between immune markers between the AHCC supplementation and placebo interventions. All analyses were performed with SPSS software (version 24) (IBM, New York, NY, USA). All p-values were two-sided, with p-values less than 0.05 considered significant.

## Results

**Figure 2:**
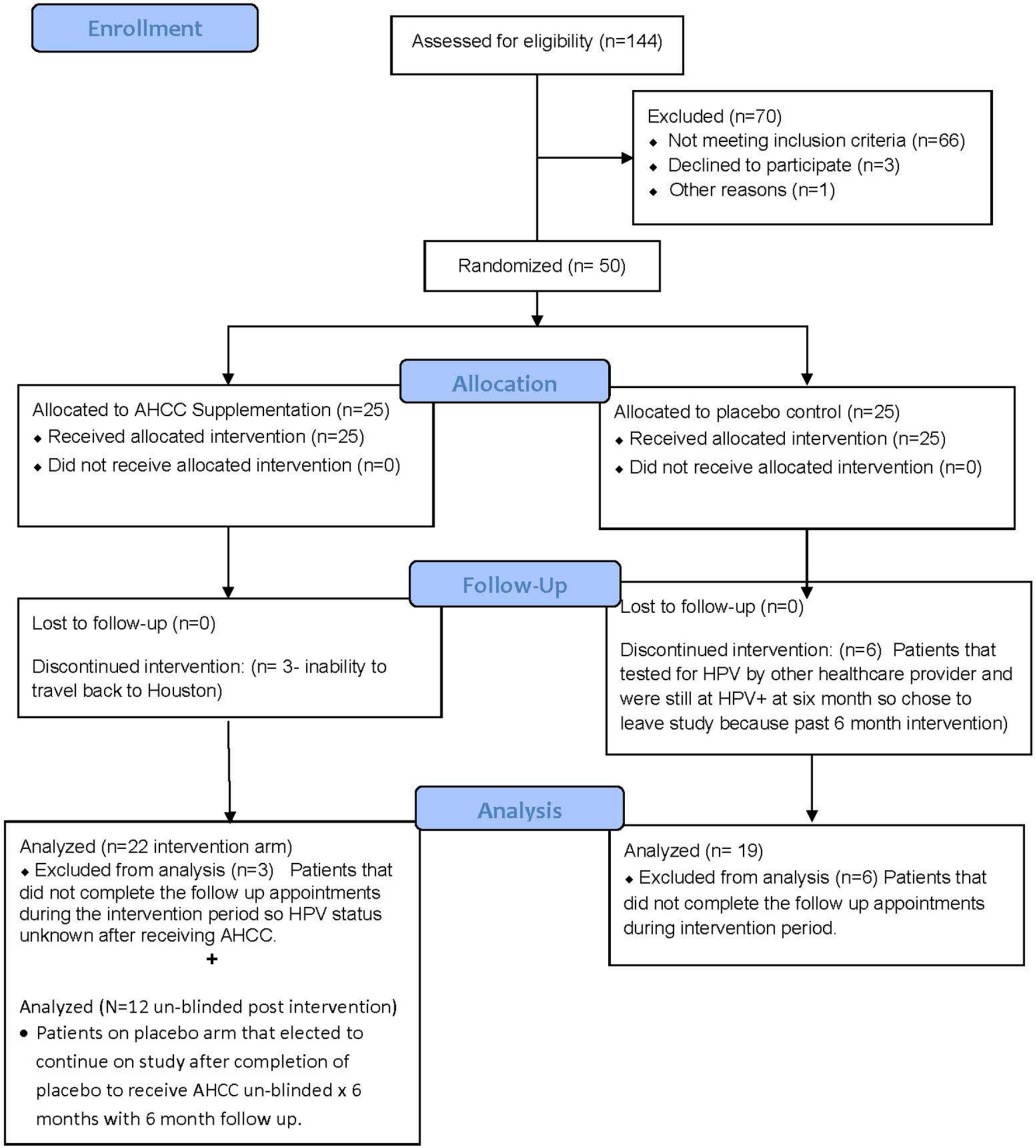
Summary of patient enrollment and analysis. This CONSORT diagram summarizes the total number of patients enrolled, completed the study, and included in data analysis.

Fifty women with HR-HPV were enrolled in this study, and 41 completed the study (**Figure 2**). All patients had confirmed high-risk HPV infection for greater than 2 years and were confirmed again at the time of enrollment. The patients in the placebo arm and AHCC supplementation were similar in age, race/ethnicity, body mass index (BMI), number of previous sexual partners, and number of current partners while in the study. Patient demographic information is summarized in **Table 2**. Fourteen (63.6%) of the 22 patients in the AHCC supplementation arm were HPV RNA/HPV DNA negative after 6 months. Nine of the 14 (64.3%) patients were still HPV negative with both HPV RNA and HPV DNA assay methods 12 months after stopping AHCC supplementation. Two of the 14 (14.3%) patients were HPV positive with both HPV RNA and HPV DNA assay methods after stopping AHCC at 12 months. Eight of the 22 (36.3%) patients in the AHCC arm were HPV positive with both HPV RNA and HPV DNA assay methods after 6 months of AHCC supplementation. On the placebo arm, two (10.5%) of 19 patients were HPV negative at 12 months. Seventeen (89.5%) patients remained HPV positive at the end of 12 months. Twelve patients completed the unblinded study, and 50% (n = 6) were HPV RNA/HPV DNA negative after 6 months of AHCC supplementation. Combining all 34 patients who received AHCC supplementation gives an overall response rate of 58.8% that cleared HPV persistent infections. These results are summarized in **Table 3**. All patients were included in the safety analysis to compare the toxicity profile of AHCC supplementation to placebo. No patients reported greater than a grade 1 (Common Terminology Criteria for Adverse Events (CTCAE) V5.0, 2018) on either placebo or the AHCC supplementation, and adverse events resolved within the first month of the study. No patients discontinued the study due to adverse events. AHCC supplementation toxicity profile was comparable to that of placebo (**Table 4**).

**Table 2:** Summary of patient demographics.

	Placebo (N = 19) Mean (SD)	AHCC (N = 22) Mean (SD)
**Age**	46.4 (± 13.5)	42.8 (± 8.9)
**Race**	15 White 3 Black 1 Asian	19 White 1 American Indian 1 Black 1 Other
**BMI (kg/m^2^)**	23.8 (± 13.4)	21 (± 11)
**Number of sexual partners**	6.3 (± 7.9)	7.8 (± 6.4)
**Number of partners in last year**	1 (± 1)	1 (± 1)

After meeting eligibility criteria, patients were randomized to either AHCC or placebo group. This table summarizes the demographic information. There were no statistical differences between the two groups.

BMI, body mass index.

**Table 3:** Summary of the HPV response.

Outcome	Placebo arm (N = 19)	Blinded AHCC arm (N = 22)	Placebo patients who went onto unblinded AHCC (N = 12)	All AHCC patients (N = 34)
**Overall response rate**	10.5% (2)	63.6 (14)	50% (6)	58.8% (20)
**CR (complete response: HPV negative after 12 months of stopping AHCC)**	10.5% (2)	40.9% (9)	50% (6)	44.1% (15)
**PR (partial response)**	NA	22.7% (5)	NA	22.7% (5)
**NR (no response)**	89.5% (17)	36.3% (8)	50% (6)	41.1 (14)

The primary endpoint of this study was confirmation of clearance of HPV by both HPV RNA and HPV DNA testing. A complete response (CR) was defined as those patients who were both HPV RNA/DNA negative after 6 months of AHCC supplementation and continued to be both HPV RNA/DNA negative 6 months off AHCC supplementation. A partial response (PR) was defined as those patients who were both HPV RNA and DNA negative after 6 months of AHCC supplementation but after 6 months off AHCC supplementation were no longer HPV DNA negative. No response (NR) was defined as those patients who remained HPV DNA positive throughout the study.

HPV, human papillomavirus; NA, Not applicable.

**Table 4:** Summary of adverse events reported in the study.

	Placebo (N = 25)	AHCC (N = 25)
**Nausea**	2 (8%)	1 (4%)
**Bloating**	1 (4%)	1(4%)
**Heartburn**	0	1 (4%)
**fatigue**	0	1 (4%)

All patients enrolled were included in safety analysis for adverse events. Overall, no study patients reported greater than grade 1 toxicity on either placebo or the AHCC supplement arm. AHCC was well tolerated compared to placebo.

**Figure 3:**
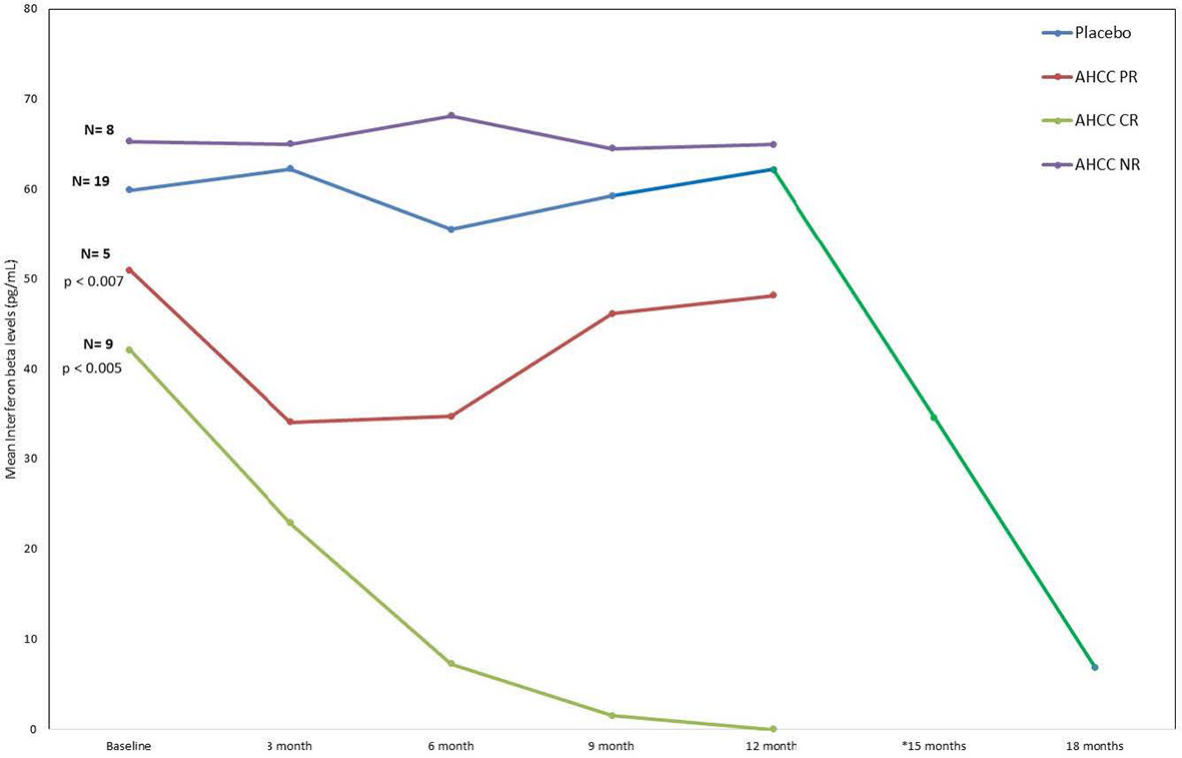
Phase II patient interferon-beta (IFN-β) response summary. This graph nicely shows how the IFN-β levels decreased in the patients who received AHCC. If the level drops below 20 pg/ml, it was associated with durable clearance of the human papillomavirus (HPV) infection. The significant drop in IFN-β (green line) at 12 months represents levels in patients who received unblinded AHCC supplementation.

**Figure 4:**
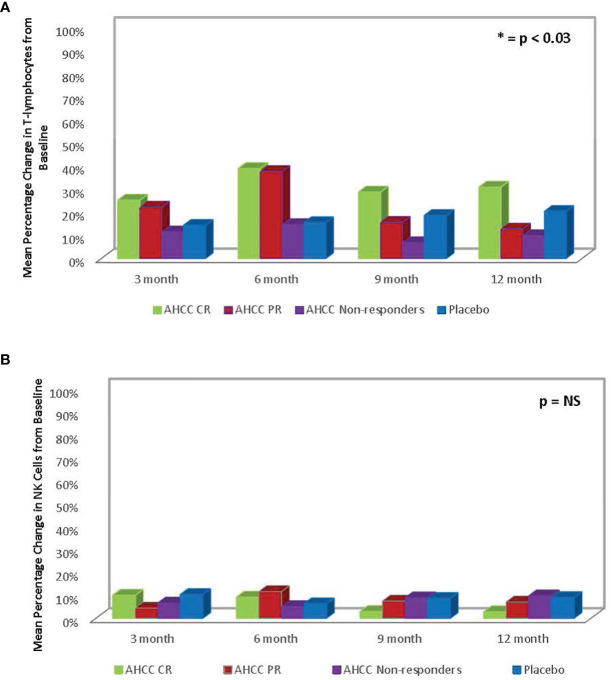
**(A)** Summary of mean percent change in T lymphocytes from baseline in phase II patients. The change in T lymphocytes correlated with clearance of human papillomavirus (HPV) infections. **(B)** Phase II patient interferon-gamma (IFN-γ) response summary. The increase in IFN-γ, a type II interferon, correlated with decrease in IFN-β, a type I interferon, and ultimately clearance of HPV infections.

ELISAs and flow analysis of total lymphocytes and NK cells performed on plasma samples at different time points from 41 patients who completed the study showed that suppression of IFN-β to less than 20 pg/ml correlated with an increase in T lymphocytes and IFN-γ and clearance of HPV infections in women who received AHCC supplementation. The mean percent change in T lymphocytes from baseline was between 25% and 45% in complete responders, which was statistically significant. There was a mean 45% increase in T lymphocytes at a 6-month time point in both complete and partial responders, whereas non-responders and placebo patients did not show a significant change in T lymphocytes. The mean percent change in NK cells was less than 10% from the baseline and was not statistically significant in all groups. These results are summarized in **Figures 3**, **4**.

## Discussion

This was the first double-blind, placebo-controlled clinical study to demonstrate that the nutritional supplement, AHCC, is effective in eliminating persistent, high-risk HPV infections with durable response in those patients who achieved an IFN-β level below 20 pg/ml. Overall, AHCC was well tolerated and had comparable adverse effects as compared to placebo.

Currently, there are limited effective options for clearing persistent HR-HPV infections, and most involve invasive procedures or local topical treatments that do not necessarily stop the HPV virus from coming back to that location or potentially other infected areas of the body such as the anus or head and neck infected epithelium. Fortunately, most patients with healthy immune systems will eliminate high-risk HPV infections within 6–18 months on their own without any interventions (16, 18). Only approximately 10% of patients usually suffer from persistent HPV infections (16, 18). There are no other systemic interventions that have been proven to have durable response to eliminate persistent HPV infections.

AHCC is a proprietary, standardized extract of cultured lentinula edodes mycelia (AHCC^®^, Amino Up, Ltd., Sapporo, Japan); the compound is primarily composed of α-glucan components as compared to most mushroom extracts, which are composed of β-glucan components. AHCC exhibits unique immune modulation to downregulate the hyper-stimulated IFN-β level resulting in negative feedback to kick-start the release of IFN-γ and T lymphocytes needed to clear chronic viral infections (17). This reset of the host immunity was demonstrated in two independent studies by Wilson and colleagues and Teijaro and colleagues, who evaluated lymphocytic choriomeningitis virus (LCMV)-persistent infections and demonstrated that suppression of chronic IFN-β signaling can reset the host immunity and enable control and clearance of persistent viral infections (19, 20). In general, β-glucan mushroom extracts are immune stimulants of the IFN-β pathway, which are helpful in supporting the immune system in acute infections but not as much in chronic viral infections. Previously, Gao and colleagues demonstrated the immunomodulating effects of AHCC in a study that showed enhanced activation of antigen (Ag) of CD4 (+) and CD8 (+) T cells as well as an increase in NK cells (10). Moreover, Roopngam and colleagues have demonstrated that β-glucans of polysaccharides from the grey oyster mushroom (*Pleurotus sajor-caju*) have potent immunomodulatory activity (21). The immunomodulation of a mushroom polysaccharide enhances the expansion of CD+/CD8+ T cells (21). The data from this clinical study also showed a significant increase in T lymphocytes in patients who were in the AHCC supplementation arm.

The human immune system begins to weaken at about 25 years of age, which perhaps could be the contributing factor to why persistent HPV infections are most often observed in women over the age of 30 or with other immunosuppressive conditions.

This phase II study confirmed findings from the two previous pilot studies that demonstrated that AHCC supplementation will modulate the host immune system to eliminate persistent high-risk HPV infections (14). While it may not help all patients, in the absence of other systemic treatments, clinicians can recommend AHCC, which is a readily available nutritional supplement that offers a good chance of clearance of persistent HPV infections.

Previously, the two pilot studies evaluating AHCC supplementation in women with persistent HR-HPV infections identified that IFN-β levels of less than 20 pg/ml correlated with the elimination of HR-HPV (17). This phase II study confirmed the correlation between suppressed IFN-β levels to less than 20 pg/ml with an increase in T lymphocytes and IFN-γ, which ultimately resulted in clearance of HPV infections in women who received AHCC supplementation. In those patients who were HPV RNA/HPV DNA negative after 6 months of AHCC supplementation but had a mean IFN-β level greater than 20 pg/ml, two remained HPV RNA negative but HPV DNA positive, and three were both HPV RNA and HPV DNA positive 3 months later after supplementation had been stopped. This identified the opportunity for future research to optimize and personalize the duration of supplementation on both HPV infection status and the target IFN-β level. In addition, the data from this study identified the potential opportunity to employ monitoring IFN-β levels, which could be used for both men and women with HPV infections. While this study did focus on women with HR-HPV infections, in the absence of effective testing tools for HPV status in men and with a safety profile comparable to placebo, the use of AHCC supplementation for men with known exposure to HR-HPV (i.e., partners of women with HR-HPV) as well as those with LR-HPV infections could consider AHCC supplementation to clear the HPV infection.

This phase II study was supported by preclinical *in vitro* studies, *in vivo* animal studies, and two pilot studies that determined the mechanism of action and confirmed the benefits of AHCC supplementation to eliminate persistent HPV infections. The major strength of this study is that even though AHCC is not a traditional drug intervention, the benefits of AHCC supplementation were held to the traditional efficacy test of a double-blind, randomized, placebo-controlled trial. Unlike many nutritional supplement studies, this study did look at specific immune markers to confirm the mechanism of AHCC modulation of host immune function. Due to limited funding resources, this study could not explore all possible mechanisms of immune modulation that may have supported clearance of the persistent HPV infections. Since the hypothesis of AHCC benefits is based upon modulation of the host immune function, specific HPV typing was not included in this study, which needs to be evaluated closer in future studies.

In conclusion, the results from this phase II study demonstrated that AHCC 3 g once daily was effective to support the host immune system to clear persistent HPV infections and was well tolerated with no significant adverse side effects reported. The duration of AHCC supplementation required beyond the first negative result needs more evaluation to optimize durable outcomes based on both HPV infection status and the target IFN-β level.

## Data Availability Statement

The original contributions presented in the study are included in the article/supplementary materials. Further inquiries can be directed to the corresponding author.

## Ethics Statement

The studies involving human participants were reviewed and approved by the University of Texas Health Sciences Center Institutional Review Board. The patients/participants provided their written informed consent to participate in this study. Written informed consent was obtained from the individual(s) for the publication of any potentially identifiable images or data included in this article.

## Author Contributions

JS served as principal investigator of the clinical trial and was responsible for study concept, protocol design, data analysis, clinical interpretation of data, writing, and finalization of the manuscript. AG, LM, and BR served as the primary research team responsible for patient screening, clinic visits, sample collection, and analysis and participated in the writing and review of the manuscript. JF served as a clinical collaborator on this study and participated in protocol design, clinical interpretation of data, and final review of the manuscript. JL served as a clinical collaborator on this study and participated in the clinical interpretation of the data and final review of the manuscript. YB served as a research collaborator participating in the interpretation of the sample of analysis, protocol design, and review of the final manuscript. RO served as a research collaborator participating in the interpretation of the sample of analysis and implications for future monitoring assays. He participated in the review and finalization of the manuscript. TB served as a clinical collaborator on this study and participated in protocol design, clinical interpretation of data, and final review of the manuscript. All authors listed have made a substantial, direct, and intellectual contribution to the work and approved it for publication.

## Funding

This study was supported by the NIH-NCI Small Grants Program for Cancer Research (1R03CA212935), which had no involvement in the study design, data collection, data analysis, and interpretation or writing of the manuscript or its publication.

## Conflict of Interest

JS was a recipient of various unrestricted research grants supporting preclinical studies on AHCC prior to 2014 from Amino Up, Ltd.

The remaining authors declare that the research was conducted in the absence of any commercial or financial relationships that could be construed as a potential conflict of interest.

## Publisher’s Note

All claims expressed in this article are solely those of the authors and do not necessarily represent those of their affiliated organizations, or those of the publisher, the editors and the reviewers. Any product that may be evaluated in this article, or claim that may be made by its manufacturer, is not guaranteed or endorsed by the publisher.
